# Weakly Supervised Collaborative Learning for Airborne Pollen Segmentation and Classification from SEM Images

**DOI:** 10.3390/life13010247

**Published:** 2023-01-16

**Authors:** Jianqiang Li, Qinlan Xu, Wenxiu Cheng, Linna Zhao, Suqin Liu, Zhengkai Gao, Xi Xu, Caihua Ye, Huanling You

**Affiliations:** 1 Faculty of Information Technology, Beijing University of Technology, Beijing 100124, China; 2 Beijing Meteorological Service Center, Beijing 100089, China

**Keywords:** deep learning, SEM, pollen identification, airborne allergic pollen

## Abstract

Existing pollen identification methods heavily rely on the scale and quality of pollen images. However, there are many impurities in real-world SEM images that should be considered. This paper proposes a collaborative learning method to jointly improve the performance of pollen segmentation and classification in a weakly supervised manner. It first locates pollen regions from the raw images based on the detection model. To improve the classification performance, we segmented the pollen grains through a pre-trained U-Net using unsupervised pollen contour features. The segmented pollen regions were fed into a deep convolutional neural network to obtain the activation maps, which were used to further refine the segmentation masks. In this way, both segmentation and classification models can be collaboratively trained, supervised by just pollen contour features and class-specific information. Extensive experiments on real-world datasets were conducted, and the results prove that our method effectively avoids impurity interference and improves pollen identification accuracy (86.6%) under the limited supervision (around 1000 images with image-level labels).

## 1. Introduction

Allergic disease, a common autoimmune disorder, is listed by the World Health Organization as one of the top three diseases to focus on in the 21st Century [1]. Large amounts of allergenic pollen wafting through the air can cause pollinosis, resulting in symptoms such as itchy eyes, tearing, and itchy nasal passages [2,3]. Globally, pollinosis has become a seasonal epidemic with considerable prevalence, and the number of patients with hay fever allergies has continued to rise in recent years [4]. Since the disease still cannot be completely controlled and is prone to recurrence [5], an efficient pollen identification system is urgently needed for pollinosis symptoms’ prevention.

Light microscopy (LM) images and scanning electric microscopy (SEM) images are mainstream tools for automatic pollen identification currently [6]. LM images are considered as a more routine method due to their simple deployment and short obtaining process. Inspired by advanced computer vision technology, LM-based approaches have been widely used for automatically recognizing airborne pollens [7,8,9,10,11,12,13,14,15,16,17,18]. For example, Li et al. [11] extracted a variety of feature descriptors, including morphological feature descriptors, Fourier descriptors, and Haralick texture feature descriptors. Daood et al. [12] proposed a CNN model with six convolutional layers and utilized a transfer learning mechanism (fine-tuned based on large natural datasets) to improve the performance of pollen identification. Sevillano et al. [13] further designed a deeper CNN model, and their experiment based on the POLEN23E dataset showed that their proposed method can achieve better results. Our team has also been actively exploring automatic pollen identification based on LM images. Wang et al. [14] presented an improved detector incorporating a self-attention mechanism to distinguish pollen and the background in LM images. Zhao et al. [15] proposed a novel pollen identification framework in a progressive manner, which perfectly mimics the manual observation process of the palynologist. However, in practice, we found that LM images suffer from the following disadvantages: (1) the detection optics used to collect the light source in LM can only focus on a fixed distance, which results in some pollen features having out-of-focus blurring; (2) all the details of the pollen grains fail to be completely captured (especially texture features) since this is particularly hindered by the low resolution of the LM scanner (usually 40× or 20×).

**Figure 1:**
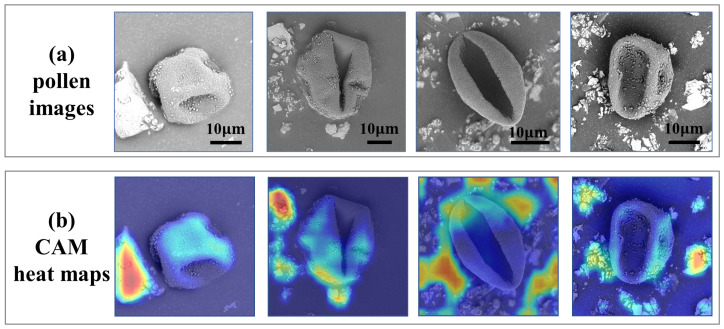
Examples of the misclassification of the pollen images and their corresponding CAM heat maps. (**a**) represents the pollen images. The large and notable object in the center is a pollen grain, and the surrounding small and irregular objects are impurities. (**b**) denotes the corresponding CAM heat maps, where the warm-colored area indicates a high-weight region of pollen recognition. Specifically, the red area represents the most-significant features in making the classification, and we can see that the network tends to focus on impurity information.

In contrast, SEM basically scans the samples with a focused beam of electrons, which can suppress the out-of-focus blurring information, and this provides a high resolution (usually 1000–3000×), so that we can see the very fine details of the pollen grains. Therefore, SEM images have exhibited significant potential to enhance the ability to accurately identify airborne pollens in an automatic manner for the palynology community [19]. Currently, some preliminary research works on SEM-based automatic pollen identification have been reported [20,21,22,23,24,25]. An early example of an SEM-image-based method was [20], in which the authors extracted the texture features to train a Fisher linear discriminant classifier. Treloar et al. [21] calculated the area, perimeter, and roundness of pollen grains, followed by a linear discriminant function to classify 12 types of pollens from SEM images. Yang et al. [22] first employed the R-CNN to detect pollen grains in SEM images, then AlexNet, GoogLeNet, and ResNet50 were trained for banana shrub and Papaver rhoeas pollen identification. To subdivide Artemisia pollen into three types, hierarchical clustering analysis on the pollen’s morphological features was carried out by Lu et al. [23]. Polling et al. [24] trained VGG16 to increase the taxonomic accuracy for Urtica and Parietaria. Based on the statistical features and histogram coefficients, MLP was trained in [25] for SEM pollen image classification. Despite the above-mentioned successes, these methods generally depend on the quality of the training image datasets. To the best of our knowledge, these methods are mainly based on purified pollen images (i.e., manually removing the noise background). However, the sample images collected in the real-world environment usually contain many impurities due to the weather, dust, and so on, which can greatly decrease the performance of the model on pollen identification. As shown in the pollen images in Figure 1, this suggests there are special shape impurities around the pollen grains. We also found from the misclassification cases of Figure 1 that the deep network tends to focus on noise information, rather than the pollen features if there are many impurities in the background. The intuitive solution is to segment the foreground (i.e., the pollen grains) and the background to eliminate the interference as much as possible; however, segmentation models need to be supervised by a large number of pixelwise labels. Since pollen particles have different shapes in the SEM images, pollen segmentation annotation is a difficult, tedious, and time-consuming task. Therefore, how to perform accurate pollen identification under limited supervision is the pivotal problem in this paper.

To solve the above problem, we propose collaborative learning for pollen segmentation and classification in a weakly supervised manner. Considering that the pollen grains in each raw image are relatively small, we firstly used a detection model in the preprocessing to detect pollen objects in the original image. For the segmentation module, we generated unsupervised segmentation labels based on the pollen’s contour features and size selection to help the initial training. For the classification module, each SEM image was masked with its pollen segmentation map as the model input to reduce the background noise and better recognize the categories. Pollen classification can benefit from accurate pollen segmentation, while class-specific information is also helpful to improve the segmentation performance. Thus, we considered collaboratively optimizing the segmentation and classification tasks. Specifically, this mechanism was built with unsupervised pollen masks and class-activation maps obtained from the segmentation and classification models, respectively. Class-activation maps refine the pollen masks so that the segmentation model can be trained with more accurate pixelwise labels. Furthermore, the classification model is able to effectively learn the semantic features and enhance the activation map extraction with accurate pollen masks. These two models were jointly trained in this collaborative learning manner to achieve the final convergence. In general, the main contributions of this paper are highlighted as follows:

- To eliminate the interference of noise in the classification model and improve the pollen classification performance, a weakly supervised collaborative learning method for pollen segmentation and classification is proposed in this paper. We input the segmented pollen region into the classification network to avoid the interference of impurities in the classification model, thus obtaining an accurate pollen activation region. This refined activation region was used to optimize the segmentation labels to improve the segmentation performance. The segmentation and classification models were collaboratively optimized to achieve the optimal performance simultaneously.
- To solve the problem of time-consuming and laborious annotation task, a weakly supervised pollen segmentation method is proposed with only the image-level annotation data. We combined digital image processing (based on the pollen contour and size selection) and gradient-weighted class-activation mapping to generate pollen segmentation pseudo labels. This method only requires image-level labels to obtain a segmentation network with superior performance.
- Numerous experiments on real-world SEM pollen images were conducted, and the results showed that our approach achieved satisfactory performance. An accuracy of 86.6% was achieved in the pollen classification task, and an MIoU of 92.47% was achieved in the segmentation task.

This paper is organized as follows. Section 2 introduces our materials and the proposed method. Section 3 describes the the experimental results and analysis. Section 4 discusses our study in detail and the future directions. The work of this paper is finally summarized in Section 5.

## 2. Materials and Methods

### 2.1. Dataset and Preprocessing

SEM images can be used to improve the performance of pollen identification in practice [20,21,22,23,24,25], since the high resolution of SEM unlocks the potential to provide the obvious differentiation of the pollen grains for the experts (as shown in Table 1). In our study, we obtained 1324 SEM pollen images in total (805 Cupressaceae, 248 Fraxinus and 271 Ginkgo) from Beijing Meteorological Center, which served as the data for our automatic pollen identification task. Our pollen samples were collected through a Hirst-type spore trap produced by Burkard Company from three sampling sites in the Dongcheng, Haidian, and Chaoyang districts of Beijing from March 1 to April 30. The trap was equipped with a built-in vacuum pump with a 2 mm/h rotational speed and with the air pumped in through a 14 mm slit at a rate of 10 L/min, which could continuously capture the airborne pollen for 24 h. The airborne pollens usually deposited on the doubled-sided carbon conductive scotch tape (Ted Pella 8 mm × 20 m) on the drum. Subsequently, the sample features were observed under the Phenom ProX SEM by the palynologists (note that it was connected to a desktop computer in order to utilize the graphical user interface), including the pollen shape, surface texture, germination groove, germination pore, etc. Finally, the digital images of the pollen grains are saved via screen capture by the palynologists for our use.

**Figure 2:**
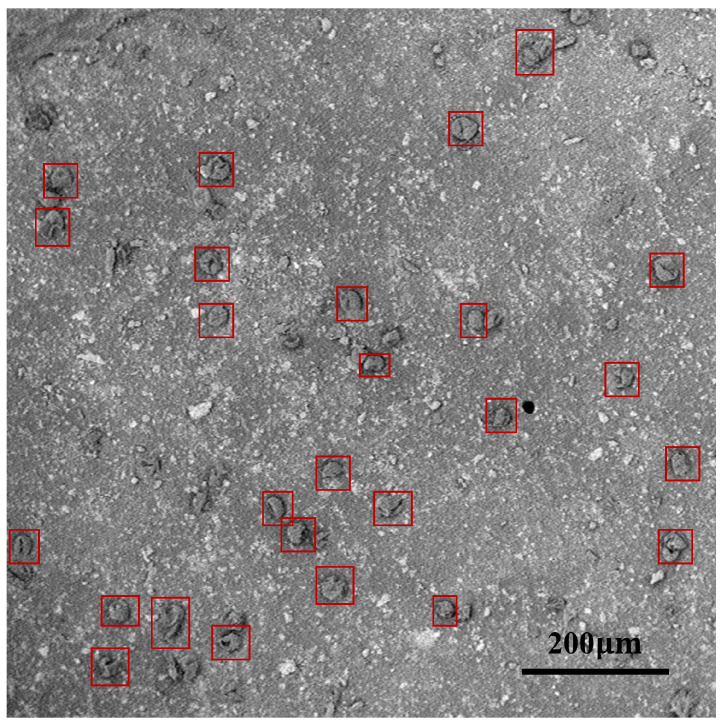
An example of airborne pollen in the scanning electron microscope (SEM) image. The figure indicates the original SEM image, and the small red boxes represent the pollen grains.

**Figure 3:**
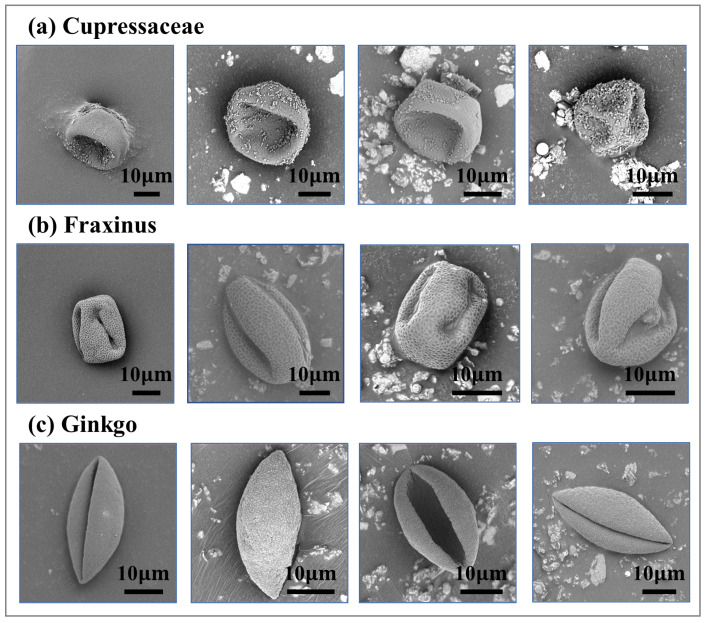
Some examples of specific pollen classes, where an image contains a single pollen grain. (**a**) Cupressaceae. The pollen grain is spherical, without germination pores, and the outer wall has small granular ornamentation. (**b**) Fraxinus. The pollen grain is long, spherical, quadrangular in polar view, and rectangular in equatorial view. (**c**) Ginkgo. The pollen grain is olive-shaped, symmetrical on both sides, wedge-shaped in long equatorial view, and round kidney-shaped in short equatorial view.

The original SEM image collected from the natural environment generally has a relatively large size. Each image inevitably includes a varied and complex blank background under such a wide field of view (as shown in Figure 2), which has a negative impact on accurate pollen identification. Thus, image preprocessing is necessary so that the original SEM images are suitable for the subsequent identification models. To this end, various object detection networks were specifically used as our image preprocessing tools. The training data with the pollen labels and the coordinate positions of the ground truth bounding boxes were additionally labeled and cross-validated by five experts. We implemented five representative object detection algorithms to train our preprocessing network: SSD [26], Fast R-CNN [27], Faster R-CNN [28], YOLOv5, and RetinaNet [29]. The detection results achieved by YOLOv5 were much better than the other detectors. In this way, we filtered out the blank background and ensured that each image only contained a single pollen grain, which laid the foundation for more accurate pollen identification in the next stage. Some preprocessed examples of pollen images are shown in Figure 3.

### 2.2. The Proposed Method

**Figure 4:**
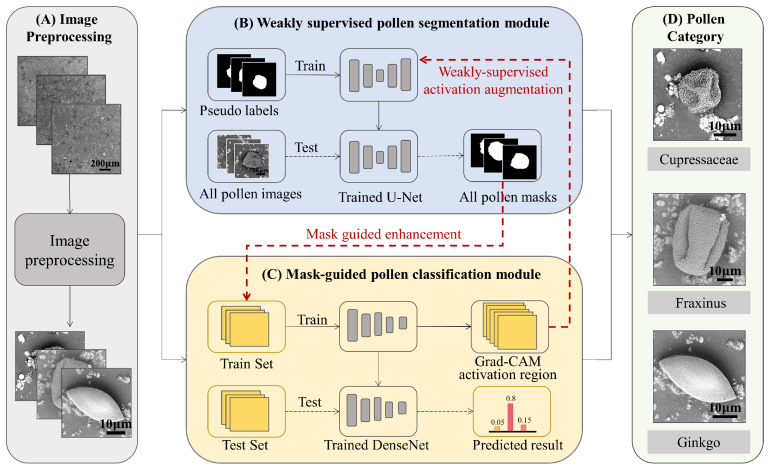
The overall network architecture of our proposed method. The gray part (**A**) represents the image preprocessing stage, which obtains an image containing only a single pollen grain through the object detection network. The blue part (**B**) represents the weakly supervised pollen segmentation module, which is dedicated to obtaining the pollen masks. These masks are fed into the classification network to guide the CNN in order to avoid the impurity interference. The yellow part (**C**) is the mask-guided pollen classification module, which aims to predict the pollen category and generate the Grad-CAM activation region. These Grad-CAM results are used to improve the quality of the pseudo labels of the segmentation network. The green part (**D**) shows the predicted pollen category.

Automated pollen segmentation and classification are two pivotal tasks in computer-aided pollen identification. The goal of the former is to remove complex impurity interference, thus allowing the extraction of the salient pollen regions, while the latter aims to accurately assign the class label to each given pollen image. However, performing accurate large-scale pixel-level annotation is time-consuming and typically requires substantial financial investments. Moreover, a reliable classification prediction heavily relies on the images with purified pollen particles, rather than the original samples, which contain complex impurities. To solve this problem, a weakly supervised pollen segmentation module and mask-guided pollen classification module are proposed to simultaneously achieve the precise region extraction and accurate category determination of pollen grains. Figure 4 provides a graphical illustration of our method. The main idea of our proposed approach is to leverage the potential benefits of segmentation and classification in a collaborative learning manner. The target activation map from the classification model was employed to refine the mask generation of the pollen grains. Besides, the boundary information generated by the segmentation model was used as the prior attention module to guide the pollen identification model to focus on the discriminative pollen features. In this way, both the segmentation and classification networks mutually transfer knowledge between each other, helping each other to enhance the learning process.

#### 2.2.1. Weakly Supervised Pollen Segmentation Module

Training a fine pollen segmentation model (e.g., U-Net [30]) usually requires a large quantity of pixelwise labeled image data. However, manual annotation is typically considered to be time-consuming and laborious, as the annotator has to draw around the irregular boundaries of the pollen regions. Given this, it is essential to find a satisfactory solution to effectively train the segmentation model. In our method, we propose a weakly supervised pollen segmentation module by combining the unsupervised pollen extraction method and the class activation augmentation driven by the deep classification network (Section 2.2.2).

**Figure 5:**
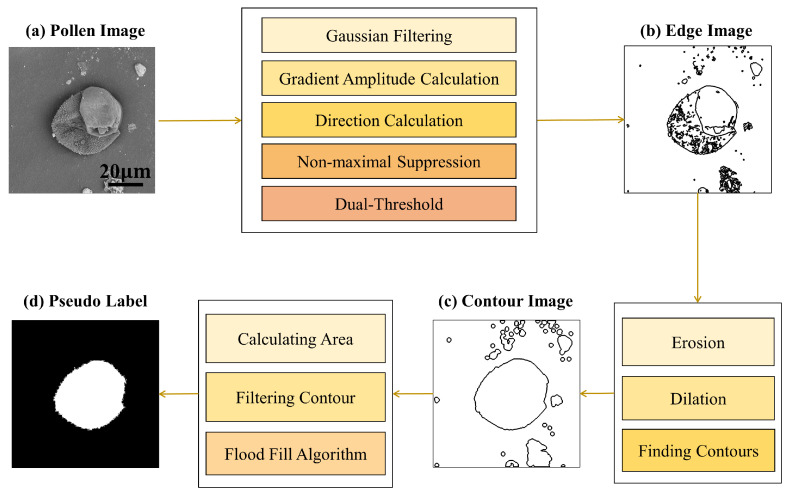
The detailed process description of unsupervised pollen extraction in our approach. (**a**) Input pollen image; (**b**) edge image after applying five different digital image processing operations; (**c**) contour image after filtering the small-scale impurities and smoothing the pollen boundaries; (**d**) the final mask (i.e., pseudo label) after impurity filtering and the flood-filling algorithm.

**Unsupervised pollen extraction:** Despite the image of individual pollen grains being obtained using data preprocessing, some impurities with special shapes remain around the pollen region. The morphological differences between the pollen and impurity prompted us to conduct purified pollen extraction based on the contour feature, as shown in Figure 5. Specifically, the contour detection is firstly performed by integrating five different digital image-processing operations, including Gaussian filtering, gradient amplitude calculation, direction calculation, non-maximal suppression, and a dual-threshold. The saliency map that can describe the boundaries of the pollen and other impurities is generated in this step. Subsequently, we employed the opening operations, including erosion and dilation, to process the saliency map with the aim of filtering the small-scale impurities while smoothing the pollen boundaries. Note that these operations solely contribute to detecting the edge of closed regions and do not significantly change the original region area. Next, the region area of each closed contour is finally calculated to separate the pollen grain from other impurities. The regions with a large area value generally indicate the potential pollen grains, since the region area of each pollen grain is considerably greater than the impurities under the field of view of the SEM. We finally applied the flood-filling algorithm to generate pseudo masks based on the saliency maps, where white color represents pollen regions and black color denotes non-pollen regions. All pseudo mask images were checked by the palynologists to remove the failed cases manually. On this basis, the candidate pseudo mask set of purified pollen grains of high quality were obtained in an unsupervised manner.

**Figure 6:**
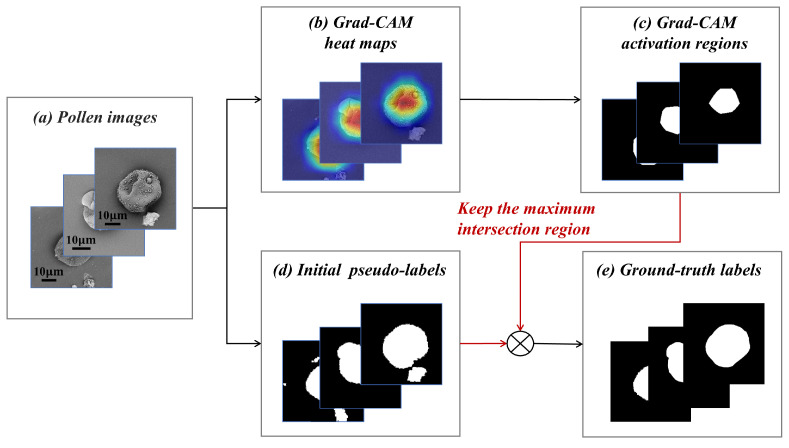
The overall pipeline of weakly supervised activation augmentation. (**a**) indicates our processed SEM pollen images. (**b**) Grad-CAM heat maps are generated from the classification network described in Section 2.2.2 by Grad-CAM. (**c**) Grad-CAM activation regions are obtained by binarizing Grad-CAM heat maps. (**d**) Initial pseudo label are obtained by unsupervised pollen extraction. (**e**) Ground truth labels are refined pollen masks used to supervise the pollen segmentation network.

**Weakly supervised activation augmentation:** The pseudo masks from the unsupervised pollen extraction offer rich boundaries of foreground object. However, this method is generally limited by the pixels of non-target objects. To optimize the pollen mask generation, we devised a strategy of combining the weakly supervised class-activation map with the unsupervised saliency map. Figure 6 illustrates the idea of our proposed method. In the upper half of Figure 6, the potential target pollen regions are inferred from the classification network by leveraging the gradient-weighted class activation mapping (Grad-CAM) method [31] described in Section 2.2.2. The class-activation mapping highlights the pixels of the discriminative region, which is useful for pollen class decision-making. We further set the specific threshold and binarized the image to extract the regions of high interest from the heat map. At this point, the localization map of the pollen grain is obtained by only using image-level labels for the supervision. It can provide the identity information of the target object to constrain the boundaries of the foreground object in the saliency map. In the bottom half of Figure 6, we selected the the maximum intersection region between the unsupervised saliency map and the target localization map as the final pollen segmentation mask. In this way, the complementary relationship between the boundary information and class information is fully utilized in order to discard the interference pixels of the non-target objects, thereby significantly improving the quality of the pseudo pollen masks.

#### 2.2.2. Mask-Guided Pollen Classification Module

The recent development of deep convolutional neural networks (DCNNs) has driven the remarkable improvements in pollen classification. Despite the great success of DCNNs, the identification performance is still limited by impurity interference from realistic environments. Thus, eliminating the noise interference is highly beneficial to improve the accuracy of pollen identification. As a matter of fact, the salient pollen masks from the weakly supervised segmentation module described in Section 2.2.1 can remove the interference in pollen images, which is considered as an excellent guide to help the classification model identify pollen grains accurately. Thus, a mask-guided enhancement is proposed to further improve the pollen classification performance of our method.

**Basic classification model:** Benefiting from the deep learning mechanism, the classical DenseNet [32], one of the representatives of the DCNN-empowered model, served as our basic classification model. The basic CNN connects each layer to every other layer in a feed-forward fashion. For each layer, the feature maps of all previous layers are used as the input, and its own feature maps are used as the input for all subsequent layers. The basic CNN can effectively alleviate the vanishing gradient problem, enhance the feature propagation and feature reuse, and greatly reduce the number of parameters. It is noted that only the original pollen images with the primary preprocessing were treated as the training dataset in this step. Additionally, the Grad-CAM [31] approach was introduced to reveal the visual decision of each given pollen image. Grad-CAM uses the gradient information to assign the importance of the spatial positions in the convolutional layer. Given a pollen image with a specific class, this method forward-propagates the image through the network to obtain a predicted score for the pollen category. The gradients were set to 0 for all classes except the desired class (which was set to 1). This signal is back-propagated to the convolution feature map we are interested in. This gradient is then used to calculate the final Grad-CAM map. Specifically, the formula of Grad-CAM is as follows:

(1) $$L_{Grad-CAM}^{c}=ReLU\left(\sum_{k}\alpha_{k}^{c}A^{k}\right)$$

where *A* is the feature layer output from the last convolutional layer; *k* is the *k*th channel in feature layer *A*; *c* is class *c*; $A^{k}$ is the data of channel *k* in feature layer *A*; $\alpha_{k}^{c}$ is the weight against $A^{k}$.

(2) $$\alpha_{k}^{c}=\frac{1}{Z}\sum_{i}\sum_{j}\frac{\partial y^{c}}{\partial A_{i,j}^{k}}$$

where $y^{c}$ is the score predicted by the network for class *C*; *A* represents the data of feature layer *A* in channel *k* with coordinates at position (i,j); *Z* equals the width × height of the feature layer.

**Mask-guided enhancement:** A general consensus has been reached that removing impurity interference is one of significant ways to boost the performance of pollen classification. In fact, the segmentation procedure (Section 2.2.1) can effectively remove the noise pixels and retain the purified pollen pixels. In this step, the mask-guided enhancement strategy is presented to further improve the pollen identification accuracy. Different from the basic classification model, we concatenated the original images with the corresponding segmented masks generated by the weakly supervised pollen segmentation as the network input. In this manner, the interference from the original images can be effectively removed, which contributes to the class-specific pollen classification.

**Collaborative learning:** The collaborative learning strategy is presented to jointly optimize the segmentation and classification tasks. During the first training round, we employed the initial segmented masks generated from the unsupervised pollen extraction and primitive class-specific images to independently train the basic segmentation and classification models. Afterwards, both tasks reinforce each other in a collaborative learning manner following the above-mentioned steps. Specifically, the images combined with the original images and pollen masks from the segmentation stage are used to train the classification models. Besides, the class-activation map indicates the potential regions of pollen grains to refine the pseudo masks that are used to train the segmentation models. The performance is improved continuously by the dual-task collaborative training.

## 3. Results

### 3.1. Experimental Settings

**Dataset settings:** The entire dataset was randomly divided into two portions called the training set and test set, 80% of which were used as the training set and the remaining 20% constituting the test set. The training set is responsible for the training of the pollen segmentation and identification models, and the test set is employed to independently evaluate the learning model. To prevent model overfitting due to the extremely limited annotated data, various data augmentation techniques were applied to enhance the training set in our experiments. The primary operations involved affine transformation (e.g., horizontal flip, vertical flip, and rotation flip) and random cropping (e.g., the intercept of the 256 × 256 pollen image cropped to 224 × 224).

**Unsupervised pollen extraction:** The Gaussian filter with a kernel size of 9 × 9 was applied to smooth the variance images and to suppress the noise. A double-threshold was adopted to obtain better edge detection, which is superior to single-threshold algorithms. Here, the two thresholds were manually adjusted to 50 and 100, respectively. The width of the detected contour line was set to 1, is considered suitable to depict the boundaries.

**Segmentation model training:** The famous U-Net architecture was chosen as the basis of out segmentation experiments. The models were trained for 100 epochs using an optimizer with a learning rate of $1\times 10^{-3}$. The cross-entropy was used as the loss function during model training. Here, this step solely detects the presence of pollen grains, separating them from the background, without distinguishing the fine-grained classes.

**Classification model training:** The DenseNet [32] model was selected as our backbone for the classification experiments. The models were trained for 100 epochs by using the Adam optimizer with a learning rate of $1\times 10^{-4}$. The cross-entropy loss function was also introduced to calculate the loss between labels and predictions.

### 3.2. Comparison with Other Methods

To prove the effectiveness of our proposed method, we compared our proposed method with other classification methods. The classical classification networks VGG [33], ResNet [34], and MobileNet [35] were selected as our comparing models in this experiment. Note that the other models were retrained over our unified training set for a fair comparison. Several common metrics were used to measure the performance, namely the precision, recall, specificity, and F1-score. The formulas are described below:

- Precision indicates the relationship between the true positive predicted values and all positive predicted values.
  (3) $$\mathrm{Precision}=\frac{TP}{TP+FP};$$
- Recall refers to the proportion of true positive predicted values on all positive samples.
  (4) $$\mathrm{Recall}=\frac{TP}{TP+FN};$$
- Specificity denotes the ability to predict negative class.
  (5) $$\mathrm{Specificity}=\frac{TN}{TN+FP};$$
- The F1-score provides an overall measure that combines precision and recall.
  (6) $$F1-\mathrm{Score}=2*(\frac{Precision*Recall}{Precision+Recall})$$

where TP and TN, respectively, represent the number of correct positive predictions and correct negative predictions. FP and FN, respectively, represent the number of incorrect positive predictions and incorrect negative predictions.

The details of the overall classification performance between our proposed method and other classification models are summarized in Table 2. As we can see from the experimental results, our approach showed the best performance with a precision of 0.864, a recall of 0.857, a specificity of 0.932 and an F1-score of 0.860. This is relatively better than the results of the other three classical models across all the evaluation metrics. In addition, Table 3 gives the classification performance of our model on every fine-grained pollen category. The recall was selected as the only evaluation indicator in this experiment, which primarily measures the ability to correctly classify all positive samples of model. The recall performance on Cupressaceae identification was close to the highest performance of the VGG model (difference = 0.03). Although the result of our proposed model on classifying Ginkgo was slightly lower than the VGG model, it showed significant superiority on Fraxinus classification (the recall was 0.063 to 0.289 higher than the other models). Overall, the experimental results were satisfactory compared with the other classification models.

### 3.3. Ablation Study

To further verify the contribution of the main modules in our approach, two ablation experiments are conducted in this section. Specifically, we designed three schemes to discuss the effectiveness of the segmentation enhancement and iteration learning modules: (1) *Baseline 1*: In this baseline, we directly trained the classification network (DenseNet) on the original pollen images. (2) *Baseline 2*: The initial pseudo masks generated from the unsupervised pollen localization were weighted on the pollen images to train the classification network. This step aimed to study whether the introduction of pollen masks could enhance the pollen identification performance. (3) *Ours*: We explored the effect of collaborative training with the segmentation-enhancement model on the classification performance. The final masks generated by the segmentation network were sent to the classification model together with the pollen image.

Table 4 shows the classification results of different methods in our ablation experiments. Compared with the basic classification model without pollen mask information, the initial pseudo masks could increase the accuracy of classification by 8.3%. *Baseline 2* also achieved better results than *Baseline 1* for the identification of individual pollen types. For example, for Ginkgo, *Baseline 2* achieved 91.8 % accuracy, 77.8 % recall, and 96.2 % specificity. This result demonstrates that pollen masks can help the deep network avoid interference from impurities. An even more significant improvement can be achieved when the classification and segmentation deep network are co-trained. The huge gain in the accuracy score of 86.6% proved that the proposed pollen segmentation model can effectively refine the pollen mask and, thus, improve the classification results. *Ours* also achieved impressive performance on single-category classification. These experimental results demonstrate that each component of our model had a positive contribution to the pollen classification task.

In addition to the improvement of pollen classification performance, we also investigated the effect of the classification model on the segmentation model. Targeting the segmentation network, we used the following evaluation indicators:

- Mean intersection over union (MIoU): The MIoU is the average of the IoU for all classes. The IoU reflects the ratio of the intersection and union of the ground truth and predicted results in each pixel class.
  (7) $$\mathrm{MIoU}=\frac{1}{C+1}\sum_{i=0}^{C}\frac{P_{ii}}{\sum_{j=0}^{C}P_{ij}+\sum_{j=0}^{C}(P_{ji}-P_{ii})};$$
- Mean pixel accuracy (MPA): The MPA is the average of the PA for all classes. The PA indicates the ratio of correctly classified pixels and the total pixels within each class.
  (8) $$\mathrm{MPA}=\frac{1}{C+1}\sum_{i=0}^{C}\frac{P_{ii}}{\sum_{j=0}^{C}P_{ij}}$$

where $P_{ii}$ represents the total number of pixels with correct prediction; $P_{ij}$ indicates the total number of pixels whose original pixel is the *i*th category, but is predicted to be the *j*th category; $P_{ji}$ indicates the total number of pixels whose original pixel is the *j*th category, but is predicted to be the *i*th category; there are C+1 categories (including *C* categories and an empty category or background category).

**Figure 7:**
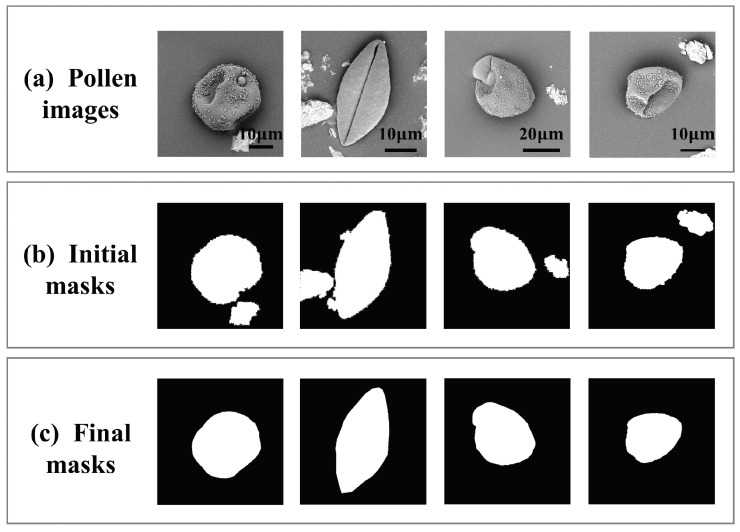
The visual comparison of the different pollen segmentation stages, including the pollen image obtained from the data preprocessing, the initial masks generated from the unsupervised pollen extraction, and the final masks after weakly supervised activation augmentation in a collaborative learning manner.

We selected three baseline segmentation models [30,36,37] to verify the impact of the collaborative optimization strategy on the segmentation model. It can be seen from Table 5 that the segmentation performances of the three baseline models were similar. After using the collaborative learning strategy, the segmentation performances of the three models achieved satisfactory performance. The experimental results showed that our collaborative learning method can not only improve the performance of pollen identification, but also have a positive impact on the segmentation network. This is mainly because the classification network gradually provides more and more accurate category information to the segmentation network, thereby avoiding non-pollen targets being segmented into pollen foregrounds. Figure 7 compares the initial pollen masks with the high-quality masks generated by our segmentation model. As we can see, the refined mask can obtain accurate pollen boundaries and discard co-occurring pixels from the impurity object, thereby significantly improving the quality of the pollen masks. For example, the second column in the figure shows that our segmentation network does not destroy the structure of the pollen grains; the remaining columns show that impurity pixels will not be mistaken for the pollen pixel category through our collaborative learning strategy. All in all, our method improved the performance of the segmentation network while improving the classification accuracy.

## 4. Discussion

**(a) Model performance:** For pollen classification, we compared our proposed method with some common classical CNN-empowered models. As we can see in Table 2 and Table 3, our proposed model performed better than other deep learning models. The F1-score of the other three models was only about 70%, much lower than the results obtained from other papers using the same model [33,34,35]. This is mainly because the data fed to the model are different. Our pollen samples were collected from real scenes in Beijing, China. The pollen image contains much impurity information, making the pollen classification task hard. In our methods, the pollen masks enable the classification model to focus more on discriminative regions instead of background or impurity information, thus strengthening the ability of pollen classification. Besides, the iterative enhancement between segmentation and classification tasks enables identification improvement. For pollen segmentation, existing methods [30,36,37] cannot accurately segment pollen grains and their performance of is poor. This is mainly due to the presence of many impurities in the real-world pollen image; they have similarities with the pollen grains with respect to the texture, shape, and other characteristics. Considering our collaborative learning strategy, the classification module can provide class-specific information for the segmentation network through the Grad-CAM localization. Therefore, a pollen segmentation network with satisfactory performance was obtained.

**(b) Practical implications:** We believe that our proposed approach has great potential for practical applications. The pseudo-label-generation mechanism for the segmentation network saves time and human resources. The iterative optimization of the segmentation and classification avoids the influence of impurities in pollen identification. In summary, our proposed method can identify pollen in SEM images with high accuracy, which helps to monitor and predict allergenic pollen in the air.

**Figure 8:**
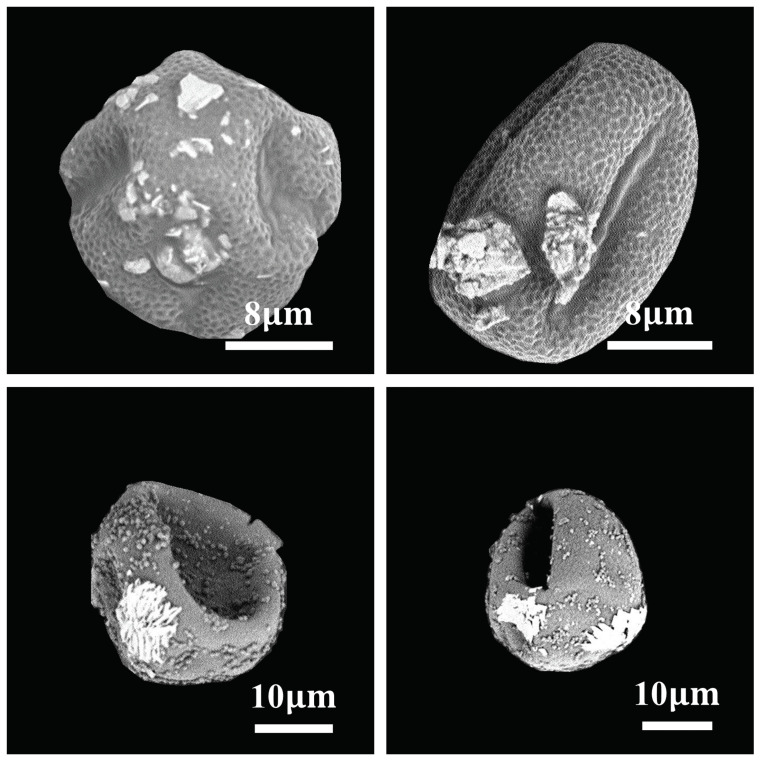
Examples of misclassification cases from our proposed method. The surfaces of these pollen grains are covered with large impurities, which hide the key features of the pollen image.

**(c) Future work:** The following improvements will be considered in the future. (1) This paper collected a relatively appropriate amount of pollen images (including three pollen types, i.e., cypress, ash, and ginkgo). We plan to expand the categories and number of pollen datasets to include as many allergenic pollen categories as possible for better monitoring of airborne allergenic pollen. Besides, samples with non-allergenic pollen and samples with non-pollen (e.g., samples with sand of comparable sizes) will also be added to our dataset to enhance the generalization ability of the model. (2) Figure 8 demonstrates some pollen images misclassified by the model. From the point of view of expert palynologists, we can conclude that the main reason is that the surfaces of these pollen grains are still covered with large impurities. The impurities hide the key features of the pollen image, causing the model to misidentify them, while the experts can determine the pollen image category by carefully observing the subtle features of the pollen images. In future work, we will consider feature fusion using contour and texture features to improve the classification performance and refine the activation maps.

## 5. Conclusions

In this paper, we proposed a weakly supervised collaborative learning approach for the segmentation and classification of SEM allergic pollen images. Our method consists of two main modules, namely weakly supervised segmentation and mask-guided classification: **weakly supervised segmentation** aims to extract pollen masks with only image-level annotations; **mask-guided classification** predicts the pollen categories with the guidance of pollen masks. Moreover, both modules mutually benefit each other in a **collaborative learning** manner: the pollen masks generated by the segmentation module are used to guide the classification network to avoid impurity interference and accurately focus on pollen grains. The pollen grain localization map generated by the classification network provides category information for the segmentation network, thereby instructing the segmentation module to effectively pick out the pollen grains. Considerable experiments on real-world SEM pollen datasets were performed, and the experimental results demonstrated the feasibility and effectiveness of our method. In the future, we will consider expanding the diversity of the data samples and further consider different pollen features, thus improving the performance of the model.

## Figures and Tables

**Table 1:** Comparison of SEM and LM.

Attribute	SEM	LM
Resolution	High (usually 6000–7000×)	Low (usually 20–40×)
Depth of field	Large depth of field	Small depth of field
Imaging quality	Sharp	Blurring
Cost	Expensive	Low
Operation	Complex	Simple
Deployment	Difficult	Convenient

**Table 2:** The comparison results on the classification performance between our proposed method and the other classical models.

Method	Precision	Recall	Specificity	F1-Score
VGG [33]	0.747	0.692	0.842	0.718
ResNet [34]	0.704	0.685	0.852	0.694
MobileNet [35]	0.756	0.667	0.832	0.709
Ours	0.864	0.857	0.932	0.860

**Table 3:** The recall performance comparison of different methods across three pollen classes.

Method	Cupressaceae	Fraxinus	Ginkgo
VGG [33]	0.968	0.712	0.396
ResNet [34]	0.923	0.486	0.646
MobileNet [35]	0.503	0.568	0.931
Ours	0.935	0.775	0.861

**Table 4:** The overall classification performance of different ablation schemes across three pollen classes.

Method	Pollen Class	Precision	Recall	Specificity	Accuracy
	Cupressaceae	0.704	0.903	0.769	
*Baseline 1*	Fraxinus	0.615	0.505	0.833	0.722
	Ginkgo	0.833	0.694	0.925	
	Cupressaceae	0.736	0.935	0.796	
*Baseline 2*	Fraxinus	0.802	0.658	0.940	0.805
	Ginkgo	0.918	0.778	0.962	
	Cupressaceae	0.858	0.935	0.906	
Ours	Fraxinus	0.843	0.775	0.946	0.866
	Ginkgo	0.892	0.861	0.944	

**Table 5:** The performance comparison of the segmentation task between the basic segmentation network and our proposed approach (CL denotes our collaborative learning method).

Method	MIoU	MPA	Accuracy
*Baseline 1* [30]	0.8291	0.9388	0.9217
*Baseline 1 + CL*	0.9247	0.9671	0.9700
*Baseline 2* [36]	0.8269	0.9380	0.9205
*Baseline 2* + CL	0.9352	0.9663	0.9742
*Baseline 3* [37]	0.8389	0.9468	0.9268
*Baseline 3* + CL	0.9289	0.9629	0.9722

## Data Availability

The data that support the findings of this study are available from the corresponding author upon reasonable request.
